# Evaluating a new *verbal working memory-balance* program: a double-blind, randomized controlled trial study on Iranian children with dyslexia

**DOI:** 10.1186/s12868-021-00660-1

**Published:** 2021-09-15

**Authors:** Mehdi Ramezani, Saeed Behzadipour, Ehsan Pourghayoomi, Mohammad Taghi Joghataei, Elham Shirazi, Angela J. Fawcett

**Affiliations:** 1 Department of Neuroscience, Faculty of Advanced Technologies in Medicine, Iran University of Medical Sciences, Tehran, Iran; 2 Mechanical Engineering Department, Sharif University of Technology, Tehran, Iran; 3 Djawad Movafaghian Research Center in Neuro-Rehabilitation Technologies, Sharif University of Technology, Tehran, Iran; 4 Cellular and Molecular Research Center, Iran University of Medical Sciences, Tehran, Iran; 5 Mental Health Research Center, Tehran Institute of Psychiatry, Iran University of Medical Sciences, Tehran, Iran; 6 Department of Psychology, Swansea University, Swansea, UK

**Keywords:** Dyslexia, Working memory, Balance, Postural control, Cerebellum, Cognitive training, Computer assisted learning

## Abstract

**Background:**

It is important to improve verbal Working Memory (WM) in reading disability, as it is a key factor in learning. There are commercial verbal WM training programs, which have some short-term effects only on the verbal WM capacity, not reading. However, because of some weaknesses in current verbal WM training programs, researchers suggested designing and developing newly structured programs that particularly target educational functions such as reading skills. In the current double-blind randomized clinical trial study, we designed a new Verbal Working Memory-Balance (VWM-B) program which was carried out using a portable robotic device. The short-term effects of the VWM-B program, on verbal WM capacity, reading skills, and postural control were investigated in Iranian children with developmental dyslexia.

**Results:**

The effectiveness of the VWM-B program was compared with the VWM-program as a traditional verbal WM training. In comparison with VWM-program, the participants who received training by the VWM-B program showed superior performance on verbal WM capacity, reading skills, and postural control after a short-term intervention.

**Conclusions:**

We proposed that the automatized postural control resulting from VWM-B training had a positive impact on improving verbal WM capacity and reading ability. Based on the critical role of the cerebellum in automatizing skills, our findings support the cerebellar deficit theory in dyslexia.

*Trial registration*: This trial was (retrospectively) registered on 8 February 2018 with the Iranian Registry of Clinical Trials (IRCT20171219037953N1).

**Supplementary Information:**

The online version contains supplementary material available at 10.1186/s12868-021-00660-1.

## Background

Developmental Dyslexia (DD) is characterized as a difficulty in learning to read accurately and fluently [1], despite adequate intelligence, conventional classroom experience, and sufficient socio-economic opportunities [2]. 5–17.5% of children in different countries suffer from DD [3, 4]. As shown below, several theories and approaches have described DD.

Many studies have supported that reading difficulties in children with DD are due to phonological deficits, explained by the phonological deficit theory [1, 5, 6]. This theory has suggested that children with DD have a specific impairment in the representation, storage, and retrieval of speech sounds (phonological awareness problems) [7]. The phonological awareness problems lead to difficulties in grapheme–phoneme decoding of the lexical items. Insufficient grapheme–phoneme decoding causes the slowing process and inadequate recognition of letters [1, 5], leading to problems in segmentation and blending. Impaired phonological representations also limit the formation of long-term phonological representations in restoring phonological (verbal) Working Memory (WM) traces [8]. Hence, children with DD usually have deficits in verbal WM in addition to problems in phonological awareness, grapheme–phoneme decoding and segmentation [9, 10]. The verbal WM engages the phonological loop component of WM and involves the temporary maintenance and manipulation of auditory-verbal information via vocal/subvocal rehearsal [11]. Extensive evidence has confirmed the existence of the verbal WM deficit in children with DD as a fundamental problem [9, 12–22]. The verbal WM deficit in these children may extend into adulthood and thereafter affect performance in all components of WM [23]. Therefore, sufficient improvement of the verbal WM capacity in children with DD is necessary [24–26].

The cerebellar deficit theory, supported by several studies, has concluded that insufficient integration of information due to mild neurobiological impairment in the cerebellum is responsible for deficits in DD [16, 27–29]. This theory has postulated that retarded or dysfunctional articulation due to a weak capacity to automatize would lead to deficient phonological representations and affect the learning of grapheme–phoneme decoding [7, 30, 31]. As mentioned above, the impaired phonological representations limit the formation of long-term phonological representations in restoring verbal WM traces [8]. Furthermore, research has confirmed the imperative role of the cerebellum in verbal WM [32]. Unlike other approaches, the cerebellar deficits theory emphasizes postural control and balance disorders in children with DD besides supporting phonological and verbal WM deficits [1, 9, 16, 27, 33]. Cerebellar insufficiency leads to difficulties in developing automatized skills [16]. Due to incomplete automaticity, balance-related problems become apparent while performing dual-tasks or more complex tasks [34]. In dual-tasks, children with DD are unable to consciously compensate for both the cognitive or motor aspects of dual-tasks [34]. Dual-task paradigm studies have shown both postural control [27, 33, 35] and cognitive performance insufficiency [27, 33] in children with DD. The postural control and cognitive demands, therefore, interact with each other in a cognitive-motor dual-task [27, 33, 35]. There is evidence that dual-task interference decreases and may even disappear while performing a dual-task condition [36]. In other words, dual-task training can improve dual-task performance [37] and this forms the motivation for the current study. Also, a cognitive-motor dual-task training program is more efficient than a single-task program to improve cognitive or motor performances [38–41] (e.g., balance performance [40, 42]). Hence, we have supposed that a training program with a dual-task condition could be more effective than a single-task program to develop the abilities in children with DD.

Numerous computerized programs including Cogmed (www.cogmed.com), Jungle Memory (www.junglememory.com), and Cognifit (www.cognifit.com) are currently used to improve the verbal WM capacity [43]. These programs are typically commercial, and several studies have taken place to examine their effectiveness [25, 43–47]. Some studies demonstrated the positive effects of these programs on reading ability [25, 44]. However, many researchers have confirmed the short-term effects of these programs only on the verbal WM capacity [43, 45–48], and in comparison to the other programs, Cogmed has larger effects on the verbal WM capacity [43]. Moreover, the current programs suffer from some weaknesses. They were not designed to teach the verbal WM explicit strategies, such as vocal/subvocal rehearsal techniques [49], and designed as a single-task training program that leads to specific-to-practice learning effects [48]. Because of these weaknesses, researchers suggested designing and developing newly structured WM programs that particularly target educational functions such as reading skills [43, 47, 50]. It seems that designing a new effective verbal WM training program for children with DD should be adapted to cover difficulties in balance and automaticity.

The current study hypothesizes that a new dual-task program that involves explicit strategies of vocal/subvocal rehearsal and targets the reading skills and balance-related performance simultaneously, would be more effective than the current programs to improve the verbal WM capacity, reading skills, or postural control. In essence, the research design compared the progress of dyslexic children who either experienced a WM battery (control group) or the same WM battery under dual-task balance conditions (intervention group) which are hypothesized to improve performance. In the present study, a dual-task, Verbal Working Memory-Balance (VWM-B) program, was designed and its short-term effects on verbal WM capacity, reading skills, and postural control were investigated in Iranian children with DD.

## Methods

### Subjects and design

**Fig. 1:**
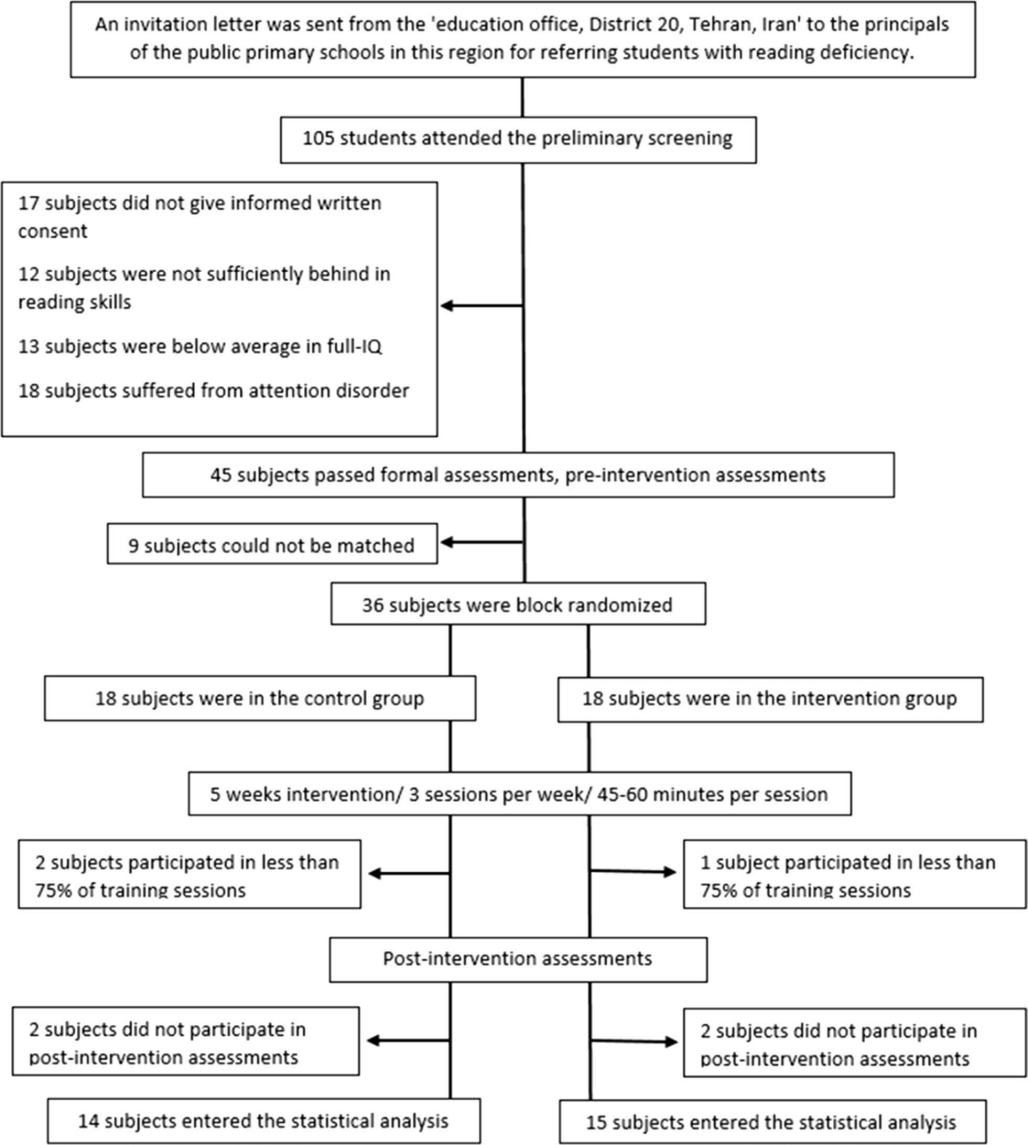
A flow chart illustrating the procedure of recruiting the participants

This quasi double-blind randomized clinical trial study was performed with a between-subjects factor ‘group’ (control group vs. intervention group) and within-subjects factor ‘time’ (measurement at pre-intervention and post-intervention) and has adhered to CONSORT guidelines (Additional file 1: Appendix S1). Data collection started in March 2018 and ended in November 2018. First, an invitation letter was sent from the ‘education office, District 20, Tehran, Iran’ to the principals of the public elementary schools in this region for referring students with reading deficiency (Fig. 1). After the invitation, children with difficulties in learning to read, reported by their teachers or those with a previous diagnosis of DD, were participated in preliminary screening. Then, the word reading efficiency (WR) and non-word reading efficiency (NWR) subtests of the validated and reliable Persian battery of normative reading tests—NEMA [51] were used to confirm the existence of DD. Children who obtained a score of 25% or less for WR and NWR subtests in the preliminary screening were included in the study [51]. Non-words are particularly important in diagnosing dyslexia for those who follow the phonological deficit hypothesis [52]. It should be noted that Iranian children begin to learn to read at the age of 4–5 years when they participate in preschool classes [53]. Preschool is an informal education course in Iran (under the supervision of the education ministry), and mandatory (at least for 1 year) before receiving a formal education course [53]. Iranian children, older than 6 years, attend the first grade of elementary school and formally learn to read [53]. With this explanation, we have requested teachers to report their students who had learning problems to read after at least 6 months of education in the first-grade. Therefore, children in the first grade of the school should have received at least 18 months of education services. However, all first-grade participants in the present study had received more than 21 months of education services.

Inclusion criteria were normal IQ, normal attention, normal vision/hearing conditions, right-handed, native-Persian language, and average socio-economic status as reported by the families. The Wechsler Intelligence Scale for Children-Fourth Edition (WISC-IV) was used to test the IQ and subjects with a WISC-IV total score ˂ 85 excluded from the study [54]. Also, the Persian version of the parent checklist of the Child Symptoms Inventory (CSI-4) was utilized to test attention (items 1 to 18 out of 97) [55, 56]. Subjects with total scores of 1–18 items ≥ 7 were excluded from the study [55–57]. Furthermore, none of the children had a history of neurological or psychiatric disorders and were taking no drugs affecting the central nervous system.

According to Fig. 1, which illustrates the procedure of recruiting participants in accordance with CONSORT guidelines [58], 36 children with DD were recruited to the study following formal diagnostic and behavioral pre-intervention assessments. However, with an approximate drop-out rate of 20%, data collected from 29 subjects entered the statistical analysis. The current study sample size is consistent with similar previous studies [44, 59–61] and is supported by Julious et al. who suggested at least 12 subjects per group in trial studies [62]. The Block randomization method in a 1:1 ratio was performed, by a computer, to allocate participants into two groups [63]. Randomization was performed in blocks of six and a block size of four to ensure a balance in sample size across groups. Also, both groups were matched by age (years), height (cm), weight (kg), full-IQ score (tested by WISC-IV [54]), and attention (tested by CSI-4 [57]), as possible confounders.

For double-blinding in the current study, children and their parents were unaware of the group to which the children had been allocated. Also, an evaluator who was not a member of the research group blinded to the subjects’ groups performed the pre-intervention and post-intervention behavioral assessments. Recording the Center of Pressure (CoP) data using a force plate (more details are given in Section “Assessments” in the “Methods”) was performed on the same day with the behavioral measures. The analyzer of CoP data was also blinded to the allocated intervention. Despite blinding children/parents and evaluator/analyzer to the allocated intervention and pre-intervention/post-intervention assessments, the participants would obviously recognize whether or not they had undertaken the training in the balance condition. Hence, the study design may be considered quasi double-blind.

Children were assessed individually at initial screening, pre-intervention, and post-intervention, separated by an average of 44 days. Outcomes of the diagnostic reading subtests, obtained from the initial screening, were used as the pre-intervention score for children who were recruited for the study. All participants in both groups completed 5 weeks, 3 days per week, one session per day, and 45–60 min per session intervention. Failing to complete a minimum of 75% of the training sessions, i.e., four out of 15 sessions, has been determined to exclude the participant’s data from the statistical analysis (all subjects, however, participated in all 15 sessions).

### Training programs

**Fig. 2:**
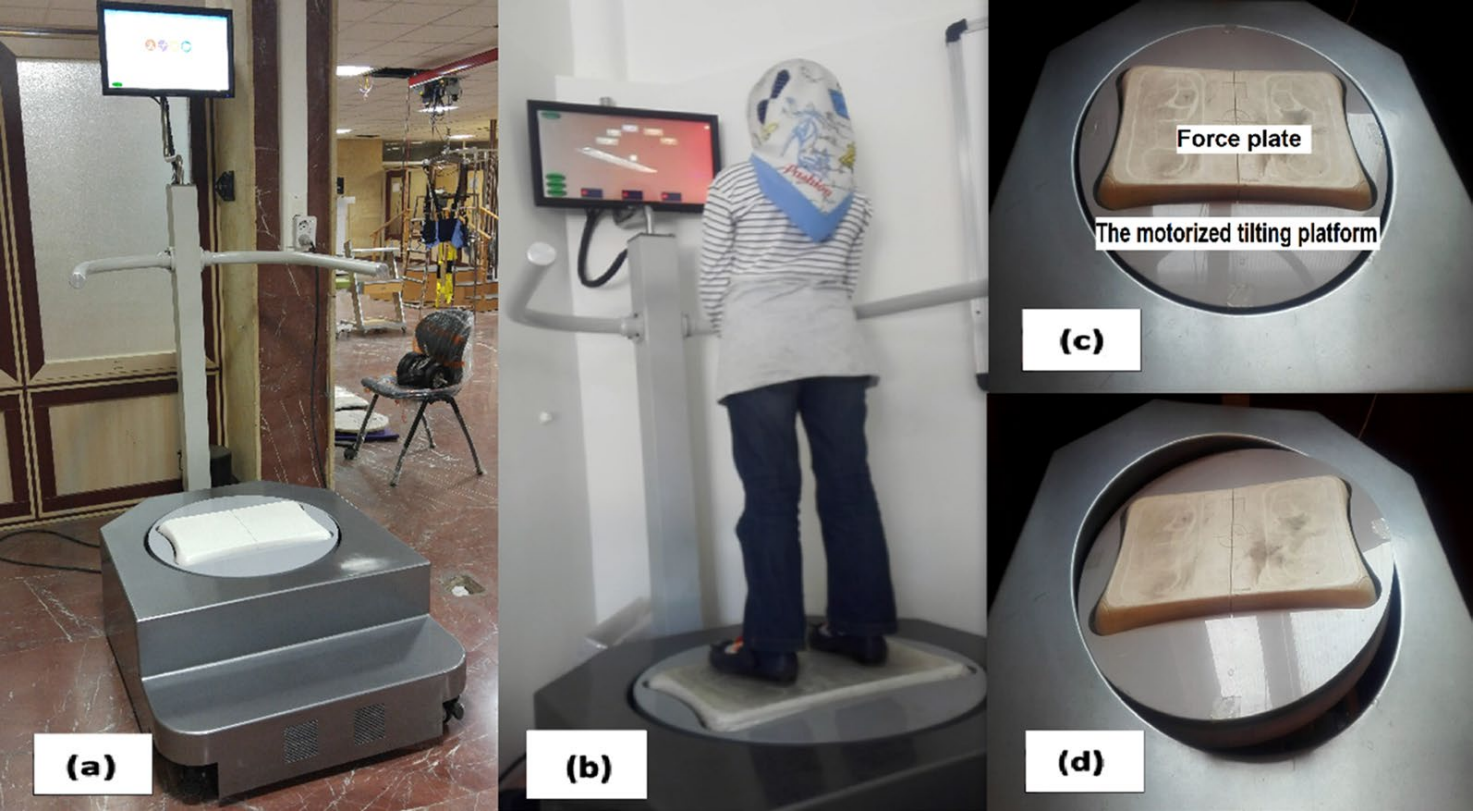
A robotic device was adopted to implement the new VWM-B training program. **a** An overview of the robot, **b** The whole setup while the subject performs a training trial, **c** The motorized tilting platform and the force plate, **d** An example of the tilting function

Based on Baddeley’s theory, verbal WM includes encoding, maintenance and manipulation of verbal information, and retrieval sub-processes [11]. Fundamental steps of programs, used in the present study, were developed with respect to this theory. In the current research, participants in control and intervention groups received training using VWM-program and VWM-B program, respectively. The VWM-program included verbal WM all sub-processes and considered as a form of the current training programs [11, 43]. A portable robotic device was also adopted and developed to perform the newly designed VWM-B program (Fig. 2a and b). The robot consisted of a platform that could be programmed to perform any desired tilting motion in the range of 0°–20° in both anteroposterior and mediolateral, or in a combination of both (Fig. 2c and d). The platform was also equipped with a force plate, with a sampling frequency of 100 Hz and an accuracy of ± 0.4 mm, to measure the CoP [64]. The setup had also a computerized interface using a 19-inch touch screen monitor, and a speaker. The computerized interface ran software that was specially designed for the proposed training program.

### VWM-program for participants in the control group

**Fig. 3:**
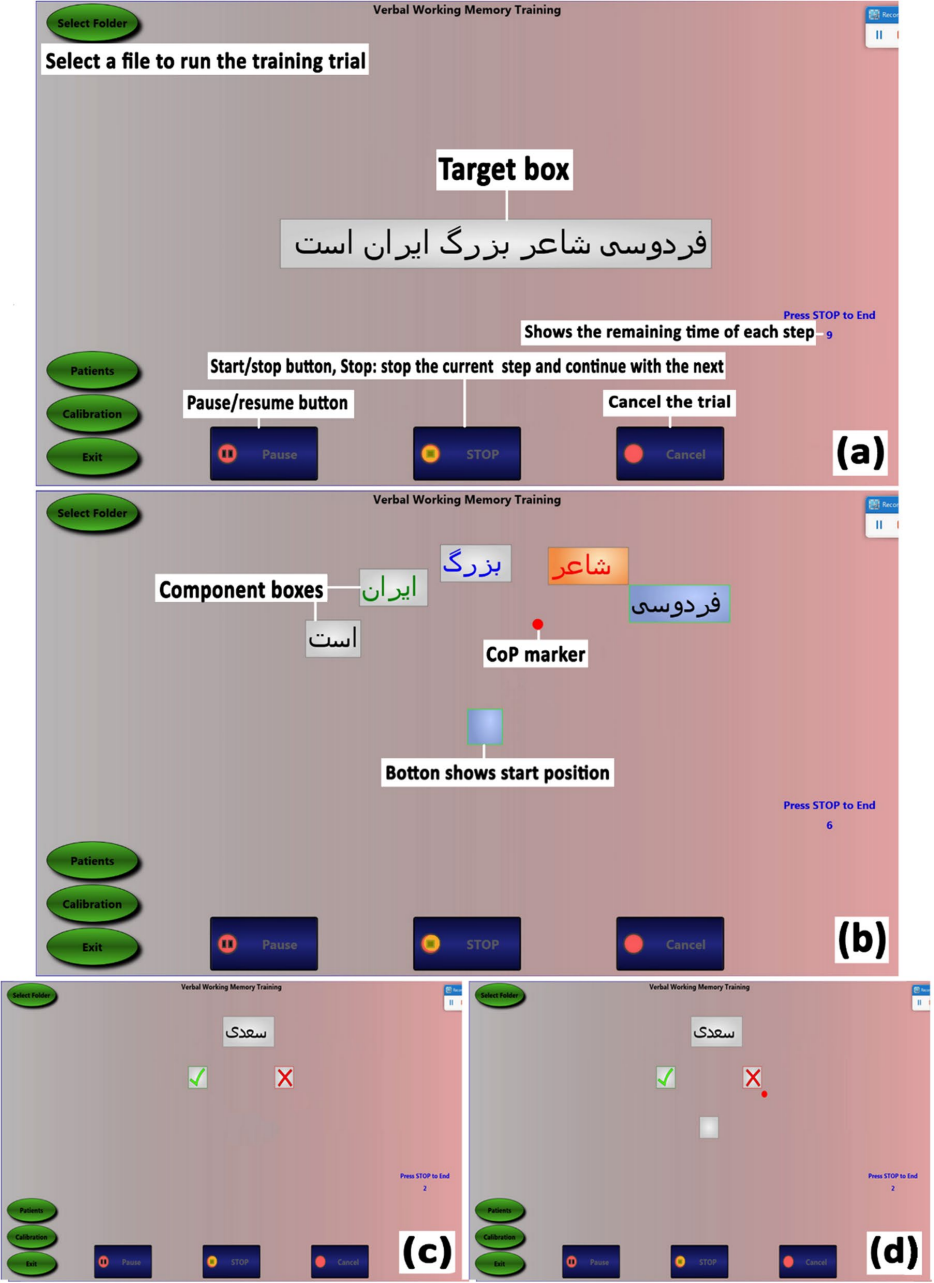
Training steps of the Verbal Working Memory (VWM) and VWM-Balance (VWM-B) programs. **a** Encoding step that is similar in both programs. **b** In the maintenance step, the target box decomposes to component boxes. The center of pressure (CoP) marker and start position button are displayed on the monitor only in the VWM-B program. In the VWM-program, a subject observes the monitor and attempts to maintain targets in the memory. However, in the VWM-B program, the maintenance step is performed in two forms: passive and active balance. In the passive state, the component box automatically hit by the CoP marker; however, in the active state, the subject actively moves the CoP marker to hit the component boxes. **c** For the retrieval step in the VWM-program, the target is shown and the user should accept or reject recalling the target. **d** In the VWM-B program’s retrieval step, the subject has to move his CoP to select the target

While training with the VWM-program, the subject sat on a chair in a relaxed mood with arms resting on the table. A 19-inch touch screen monitor ran the software, which was specially designed for the proposed training program, and a speaker was used to recite the words. As mentioned above, each training trial of the VWM-program includes all three sub-processes (encoding, maintenance, and retrieval steps) of the verbal WM. Each trial began 3 s after touching the start button on the monitor (see the start/stop button in Fig. 3a). For the encoding step, the target, which could be a word, a series of words, or a statement, written inside a box (target box), was shown on the monitor for 10 s (Fig. 3a). At the same time, the target was recited by playing a pre-recorded voice on the computer. For the maintenance step, the target was decomposed to its components (sentence to its words or word to its letters) and shown on the monitor inside separate boxes (component boxes) for 10 s (Fig. 3b). Finally, for the retrieval step, twice as many boxes, which included the practiced components and new ones appeared on the monitor (Fig. 3c). The participant had 10 s to select and touch the boxes, which had appeared and been recited as a component of the target.

### VWM-B program for participants in the intervention group

As stated above, the newly designed VWM-B program has been performed using the robotic device (Fig. 2). Like the VWM-program, each training trial in the VWM-B program includes all three steps of verbal WM. The difference is that balance movements have been combined with the maintenance and retrieval steps of verbal WM. Before the training session, the amplitude of the CoP movement was calibrated to each participant's limit of stability for safety [65]. Participants’ standing condition was controlled for uniformity among subjects. The feet position on the platform was the same for all participants, with an approximate distance of 10 cm between the feet. Also, the monitor was located at eye level, with a distance of approximately 50 cm (Fig. 2b).

For the encoding step, similar to the VWM-program, a trial began 3 s after touching the start button on the monitor, and the main target box appeared on the monitor for 10 s (Fig. 3a). Then, the component boxes appeared on the screen. In addition to the component boxes, a red circle (CoP marker) also appeared on the screen (Fig. 3b). This circle represented the position of the subject's CoP and was used to introduce balance tasks to the program.

As a new method, training the maintenance and manipulation of information was performed in two forms: passive and active balance. In the passive state, the motorized moving platform underneath the subject’s feet was tilted and the CoP marker was correspondingly moved toward the component boxes implying a passive exercise (see Figs. 2b, d, and 3b). After the component box was hit by the CoP marker, the participant had 10 s to recite the word inside the box. Then, the platform and the CoP marker returned to the start position (Fig. 3b). This procedure was repeated for all component boxes in the correct order. In the active state, the platform had no tilting motion, and the subject had to actively move his CoP towards the component boxes using ankle/hip strategies. After hitting each component box, the participant attempted to read the word aloud without time limitation. Following reading the word, he returned to the start position and repeated the procedure for all component boxes. In sum, the maintenance step of the VWM-program was 10 s. However, the maintenance step in the VWM-B program included two phases: (1) the passive state that limited for 10 s, and (2) the active state that the subject had free time to read the word. Retrieving information in the VWM-B program was similar to the VWM-program except that the subject had 10 s to select the response option using his CoP movements (Fig. 3d).

In sum, the encoding step is similar in both programs. Also, the target box decomposes to component boxes in the maintenance step of both two programs. In the VWM-program, a subject observes the monitor and attempts to maintain targets in the memory. However, in the VWM-B program, the maintenance step is performed in two forms: passive and active balance. In the passive state, the component box is automatically hit by the CoP marker; however, in the active state, the subject actively moves the CoP marker to hit the component boxes. For the retrieval step in the VWM-program, the target is shown and the user should accept or reject recalling the target. However, in the VWM-B program's retrieval step, the subject has to move his CoP to select the target.

To make training trials progressively more difficult in both programs and selecting suitable words to practice, we considered factors that impacted on the verbal WM, including phonological similarity, word length, articulatory suppression, and irrelevant sound effects [11]. Training trials for sessions were determined based on the subject’s capacity in verbal WM and reading. In the maintenance step of the VWM-B program, the main target box was decomposed to 2–9 component boxes (Fig. 3b). In the passive state, the duration of the marker/platform displacement was adjustable between 3 and 10 s. Also, to further the balance challenge, the component boxes were placed at different distances from the start position button (Fig. 3b).

### Assessments

Five subtests of the NEMA [51] were used to confirm the existence of DD and provide the pre-intervention and post-intervention assessments. The selected NEMA subtests included the WR, NWR, phoneme deletion, text comprehension, and word chain. Also, the oldest and widely used measure of Forward Digit Span (FDS) was employed to test the verbal WM capacity [66, 67].

The Stroop color-word test was used to measure changes in selective attention [68–70]. This validated test includes three components. In the color-naming component, the subject is asked to name the color of 176 bars with colors of red, blue, green, and yellow. In the word-naming component, the subject is asked to read a series of color words including 176 words with colors of red, blue, green, and yellow. Here, the subject reads the word by ignoring its color. In the color-word component, the subject is asked to name the color of words presented in the word-naming component by ignoring their printed form [68, 71]. In the current research, the time for each component was recorded. Then, the color-word interference was calculated as *the time of the third component minus the time of the second component* [71].

To assess the postural stability, the CoP data were collected with a portable customized force plate [64]. The force plate was linked via a cable connection to the computer. Data were collected at a sampling rate of 100 Hz [64]. Recording of the CoP data was performed in a quiet stance with two conditions eyes open and closed. For each condition, two recordings, with a duration of 70 s, was recorded and the mean value of parameters were used [72]. In eyes open, the subject’s gaze was fixed at a cross mark that was placed on a wall four meters ahead [73]. During the test, participants stood without shoes, with arms folded across the chest. Feet position on the force plate were also marked for inter-trial repeatability. They were instructed to remain still and relaxed in the given stance.

The CoP parameters are suitable to measure postural control in children with DD [29, 74]. We analyzed the validated parameters of the CoP including the surface area (ellipse with 95% of CoP excursions), the length (the path length of the CoP), the mean velocity, and the standard deviation in anteroposterior and mediolateral directions [72, 73, 75]. These are efficient measures of the CoP spatial variability and good indices of the amount of neuromuscular activity required to regulate postural control [29, 76]. After removing the first and the last 5 s of data, the signals were low-pass filtered using a 4th order Butterworth filter with a cut-off frequency of 10 Hz [65, 73, 77]. Data was analyzed using Matlab R2016b (MathWorks, MA, USA).

### Analysis

The normality was tested by Shapiro–Wilk [78]. Depending on the distribution of variables, independent *t*-test for parametric variables, Mann–Whitney *U*-test for non-parametric variables, and chi-square test for categorical variables were used to compare groups at baseline (*α* = 0.05). The mean (SD) for quantitative variables and the absolute frequency (%) for qualitative variables were reported. In the current study, both two groups had received the intervention. Hence, the mixed between-within ANOVA analyses were used to verify the treatment effects over time [79]. Significant interactions (*p* < 0.05) and effect size, with partial eta squared (η_p_^2^), were reported [79]. The η_p_^2^ is in the family of correlational effect size [80] and is most useful for comparing effect sizes in mixed designs [81]. There are no agreed standards for how to interpret an effect size. Nevertheless, 0.2 is considered a small improvement, 0.5 medium, and 0.8 and above large [80]. Using mixed ANOVA, the effects of training programs on the verbal WM (tested by FDS), reading skills (tested by WR, NWR, phoneme deletion, text comprehension, and word chain subtests of the NEMA), attention (tested by Stroop test), and CoP parameters including the surface area, the length, the mean velocity, and the standard deviation in anteroposterior and mediolateral directions were analyzed. Pearson’s correlations between the entire sample gain scores (i.e., the difference between scores in the measurements at pre-intervention and post-intervention) were also reported to explore relationships between the clinical and CoP measures. The SPSS 21 (SPSS Statistics, version 11, IBM, and Armonk, New York, USA) was used to analyze the data.

## Results

The control group contained 14 children with DD, with a mean (SD) age of 8 (1.22) years. The Intervention group included 15 children with DD, with a mean (SD) age of 8 (0.96) years. The hypotheses of normality for height, weight, and full-IQ scores were accepted (*p* > 0.05), and the independent *t*-test showed no significant between-group difference at baseline scores. However, the hypotheses of normality for age and CSI-4 scores were rejected (*p* < 0.05), and the Mann–Whitney *U*-test showed no significant between-group difference at baseline scores. As a result, two groups were homogenized (*p* > 0.05) for age, height, weight, full-IQ, and attention, as possible confounders (Table 1). Further information about the demographic characteristics of participants is shown in Table 1. Also, no significant difference (*p* > 0.05) was found for baseline scores of clinical and CoP measures (see *t*-test outcomes in Table 2). Table 2 also presents the alteration in the mean (SD) of clinical and CoP measures outcomes after the intervention.

**Table 1:** Demographic characteristics

Demography	CG (n = 14)	IG (n = 15)	Total (N = 29)	Group differences (*p*-value)
Mean (SD)				
Age (years)	8 (1.22)	8 (0.96)	8 (1.12)	u = 74.50 (0.158)
Height (cm)	129 (11.65)	119 (9.93)	124 (11.58)	t = − 2.28 (0.277)
Weight (kg)	31.21 (8.68)	26.20 (6.18)	28.62 (7.79)	t = − 1.78 (0.230)
WISC-IV (total score)	91.86 (4.11)	95.07 (6.56)	93.52 (5.66)	t = 1.59 (0.820)
CSI-4, parent checklist (total scores of 1 to 18 items)	2.59 (2.14)	3.41 (2.61)	3.00 (2.39)	u = 82.50 (0.316)
Frequency (%)				
Gender				
Boy	5 (35.70)	3 (20.00)	8 (27.60)	χ^2^ = 0.89 (0.344)
Girl	9 (64.30)	12 (80.00)	21 (72.40)	
Disability				
Reading	5 (35.70)	2 (13.30)	7 (24.10)	χ^2^ = 5.73 (0.126)
Reading/writing	4 (28.60)	10 (66.70)	14 (48.30)	
Reading/math	0 (0.00)	1 (6.70)	1 (3.40)	
Reading/writing/math	5 (35.70)	2 (13.30)	7 (24.10)	
School grade				
First	6 (42.90)	8 (53.30)	14 (48.30)	χ^2^ = 5.70 (0.058)
Second	1 (7.10)	5 (33.30)	6 (20.70)	
Third	7 (50.00)	2 (13.30)	9 (31.00)	

No significant differences were found on demographic data between children with dyslexia in the control and intervention groups

*WISC-IV* Wechsler intelligence scale for children-fourth edition, *CSI-4* child symptoms inventory, *CG* control group, *IG* intervention group

**Table 2:** Mean (standard deviation) of the clinical and center of pressure measures

Outcomes	CG (n = 14)		IG (n = 15)		Total (N = 29)		T (*p*-value)
	Pre	Post	Pre	Post	Pre	Post	
Clinical measures							
FDS	4.28 (0.91)	5.43 (1.39)	4.07 (1.03)	7.60 (1.72)	4.17 (0.96)	6.55 (1.90)	− 0.61 (0.998)
SCWT	180.79 (56.97)	146.21 (66.91)	144.87 (84.20)	83.27 (85.13)	162.21 (73.38)	113.65 (82.02)	− 0.35 (0.198)
WR	40.14 (30.91)	55.93 (27.87)	43.67 (26.42)	118.80 (67.29)	41.97 (28.21)	88.45 (60.39)	0.33 (0.303)
NWR	12.07 (4.32)	14.35 (4.60)	11.47 (5.39)	22.33 (8.56)	11.76 (4.83)	18.48 (7.93)	− 0.33 (0.300)
PD	13.36 (9.86)	17.14 (10.11)	15.67 (9.24)	25.40 (6.83)	14.55 (9.44)	21.41 (9.40)	0.65 (0.606)
TC	3.21 (1.67)	4.35 (1.94)	3.93 (1.33)	6.13 (1.18)	3.59 (1.52)	5.27 (1.81)	1.27 (0.266)
CW	12.50 (7.73)	17.21 (8.63)	10.27 (7.01)	29.13 (15.9)	11.34 (7.32)	23.37 (13.61)	− 0.81 (0.558)
CoP measures							
QO-L (cm)	113.56 (40.50)	126.03 (44.85)	107.82 (23.09)	95.77 (26.03)	110.59 (32.20)	110.38 (39.09)	− 0.47 (0.647)
QO-A (cm^2^)	7.11 (4.56)	8.62 (4.97)	7.97 (3.50)	5.35 (2.77)	7.56 (4.00)	6.93 (4.25)	0.57 (0.576)
QO-MV (cm/s)	1.90 (0.67)	2.10 (0.74)	1.80 (0.38)	1.60 (0.44)	1.84 (0.54)	1.84 (0.65)	− 0.47 (0.647)
QO-AP (SD)	0.61 (0.22)	0.72 (0.28)	0.66 (0.18)	0.53 (0.11)	0.64 (0.20)	0.62 (0.23)	0.72 (0.480)
QO-ML (SD)	0.55 (0.18)	0.57 (0.19)	0.61 (0.18)	0.48 (0.15)	0.58 (0.18)	0.52 (0.17)	0.85 (0.405)
QC-L (cm)	142.95 (39.91)	135.72 (35.81)	138.39 (30.19)	128.43 (35.39)	140.59 (34.12)	131.95 (35.15)	− 0.35 (0.729)
QC-A (cm^2^)	8.72 (5.16)	9.52 (5.63)	9.14 (4.23)	7.76 (4.34)	8.93 (4.62)	8.61 (4.99)	0.24 (0.809)
QC-MV (cm/s)	2.38 (0.65)	2.26 (0.59)	2.31 (0.50)	2.12 (0.58)	2.34 (0.57)	2.19 (0.58)	− 0.35 (0.729)
QC-AP (SD)	0.70 (0.24)	0.81 (0.27)	0.77 (0.23)	0.67 (0.16)	0.73 (0.23)	0.74 (0.23)	0.83 (0.415)
QC-ML (SD)	0.59 (0.24)	0.56 (0.23)	0.59 (0.15)	0.53 (0.17)	0.59 (0.19)	0.55 (0.19)	− 0.01 (0.996)

*FDS* forward digit span, *SCWT* Stroop color-word test, *WR* word reading, *NWR* non-word reading, *PD* phoneme deletion, *TC* text comprehension, *CW* chain word, *CoP* center of pressure, *QO* quite stance-open eyes, *L* length, *A* area, *MV* mean velocity, *AP* anterior–posterior, *ML* medial–lateral, *QC* quite stance-closed eyes, *CG* control group, *IG* intervention group, *Pre* pre-intervention, *Post* post-intervention

The mixed ANOVA was used to assess the impact of training programs on participants’ scores on the FDS, NEMA subtests, and CoP measures (for more information see Table 3). The time main effect was significant for outcome measures of the FDS, reading subtests, and the mean velocity parameter of the CoP in the eyes-closed condition. These results indicate the alteration in scores after the intervention, regardless of the participants’ group. The group main effect was significant for outcome measures of the FDS, WR, and text comprehension subtests of the NEMA. These results demonstrate that scores of these measures changed in groups, regardless of the time effect. The time × group interaction was also significant for all measures of the FDS, reading subtests, and CoP parameters, except the length, mean velocity, and the standard deviation of mediolateral direction in the eyes-closed condition. When the time × group interaction is significant, it means that there are differences between the two groups over time.

**Table 3:** Outcomes of mixed between-within ANOVA analyses

Outcomes	Time^a^			Group^a^			Interaction		
	f	*p*-value	η_p_^2^	f	*p*-value	η_p_^2^	f	*p*-value	η_p_^2^
	(1–27)			(1–27)			(1–27)		
Clinical measures									
FDS	103.15	** < 0.001**	0.79	5.17	**0.031**	0.16	26.95	** < 0.001**	0.5
SCWT	58.05	** < 0.001**	0.68	3.35	0.078	0.11	4.58	**0.041**	0.15
WR	47.89	** < 0.001**	0.64	5.47	**0.027**	0.17	20.4	** < 0.001**	0.43
NWR	33.94	** < 0.001**	0.56	3.66	0.067	0.12	14.45	**0.001**	0.35
PD	69.08	** < 0.001**	0.72	2.61	0.118	0.09	13.37	**0.001**	0.33
TC	154.79	** < 0.001**	0.85	4.94	**0.035**	0.16	15.48	**0.001**	0.36
CW	103.15	** < 0.001**	0.6	2.15	0.154	0.07	14.47	**0.001**	0.35
CoP measures									
QO-L (cm)	0	0.972	0	2.45	0.13	0.08	4.48	**0.044**	0.14
QO-A (cm^2^)	1.37	0.252	0.05	0.73	0.401	0.03	19.11	** < 0.001**	0.41
QO-MV (cm/s)	0	0.993	0	2.41	0.132	0.08	4.39	**0.046**	0.14
QO-AP (SD)	0.07	0.79	0	1.1	0.305	0.04	10.05	**0.004**	0.27
QO-ML (SD)	3.97	0.056	0.13	0.11	0.747	0	9.25	**0.005**	0.26
QC-L (cm)	4.07	0.054	0.13	0.23	0.635	0.01	0.1	0.752	0
QC-A (cm^2^)	0.31	0.584	0.01	0.15	0.704	0.01	4.52	**0.043**	0.14
QC-MV (cm/s)	4.51	**0.043**	0.14	0.27	0.608	0.01	0.18	0.674	0.01
QC-AP (SD)	0.04	0.842	0	0.14	0.711	0.01	8.34	**0.008**	0.24
QC-ML (SD)	3.41	0.076	0.11	0.05	0.83	0	0.45	0.511	0.02

Bolded values indicate statistically significant *p*-values (*p* < 0.05)

*FDS* forward digit span, *SCWT* Stroop color-word test, *WR* word reading, *NWR* non-word reading, *PD* phoneme deletion, *TC* text comprehension, *CW* chain word, *CoP* center of pressure, *QO* quite stance-open eyes, *L* length, *A* area, *MV* mean velocity, *AP* anterior–posterior, *ML* medial–lateral, *QC* quite stance-closed eyes

^a^Between-subjects factor ‘Group’ = control group vs. intervention group, and within-subjects factor ‘Time’ = measurement at before and after intervention

Pearson's correlation coefficients of the entire sample are reported in Table 4. Some CoP measures in eyes-open and closed conditions were correlated with FDS. The CoP measures, especially in the eyes-open condition, were correlated with reading subtests. The FDS was correlated with reading subtests. The Stroop test was also correlated only with the WR subtest of NEMA.

**Table 4:** Pearson correlation between measures r (*p*-value)

Outcomes	FDS	SCWT	WR	NWR	PD	TC	CW
Clinical measures							
SCWT	− 0.28 (0.148)	–	–	–	–	–	–
WR	0.70** (**˂0.001**)	−0.36* (**0.045**)	–	–	–	–	–
NWR	0.61** (**˂0.001**)	− 0.13 (0.495)	0.51** (**0.005**)	–	–	–	–
PD	0.41* (0**.027**)	− 0.16 (0.410)	0.46* (**0.013**)	0.41* (**0.028**)	–	–	–
TC	0.36 (0.056)	− 0.20 (0.301)	0.34 (0.069)	0.45* (**0.015**)	0.47** (**0.010**)	–	–
CW	0.60** (**0.001**)	− 0.25 (0.184)	0.87** (**˂0.001**)	0.41* (**0.026**)	0.39* (**0.038**)	0.35 (0.060)	–
CoP measures							
QO-L (cm)	− 0.25 (0.186)	0.05 (0.814)	− 0.39* **(0.035)**	− 0.26 (0.167)	− 0.50** (**0.006**)	0.01 (0.970)	− 0.26 (0.179)
QO-A (cm^2^)	− 0.52** (**0.004**)	0.20 (0.303)	− 0.58** (**0.001**)	− 0.42* **(0.024)**	− 0.47* **(0.010)**	− 0.38* (0.045)	− 0.44* (0.017)
QO-MV (cm/s)	− 0.25 (0.187)	0.05 (0.816)	− 0.39* **(0.036)**	− 0.26 (0.169)	− 0.50** (**0.006**)	0.016 (0.936)	− 0.26 (0.181)
QO-AP (SD)	− 0.29 (0.130)	0.15 (0.433)	− 0.42* **(0.023)**	− 0.18 (0.342)	− 0.33 (0.079)	− 0.22 (0.253)	− 0.35 (0.066)
QO-ML (SD)	− 0.60** (**0.001**)	0.06 (0.739)	− 0.56** (**0.002**)	− 0.52** (**0.004**)	− 0.45* **(0.014)**	− 0.45* **(0.015)**	− 0.46* **(0.011)**
QC-L (cm)	− 0.18 (0.351)	0.13 (0.495)	− 0.33 (0.077)	0.06 (0.759)	− 0.18 (0.345)	0.10 (0.618)	− 0.31 (0.106)
QC-A (cm^2^)	− 0.47** (**0.010**)	0.17 (0.368)	− 0.26 (0.150)	− 0.16 (0.415)	− 0.25 (0.197)	− 0.04 (0.849)	− 0.26 (0.177)
QC-MV (cm/s)	− 0.20 (0.307)	0.14 (0.486)	− 0.36 (0.053)	0.05 (0.806)	− 0.19 (0.319)	0.09 (0.647)	− 0.31 (0.107)
QC-AP (SD)	− 0.29 (0.125)	0.31 (0.099)	− 0.31 (0.097)	− 0.16 (0.423)	− 0.55** (**0.002**)	− 0.21 (0.267)	− 0.29 (0.127)
QC-ML (SD)	− 0.41* **(0.028)**	− 0.17 (0.388)	− 0.22 (0.254)	− 0.15 (0.445)	− 0.00 (0.986)	0.06 (0.744)	− 0.17 (0.380)

Bolded values indicate statistically significant *p*-values (*p* < 0.05)

*FDS* forward digit span, *SCWT* Stroop color-word test, *WR* word reading, *NWR* non-word reading, *PD* phoneme deletion, *TC* text comprehension, *CW* chain-word, *CoP* center of pressure, *QO* quite stance-open eyes, *L* length, *A* area, *MV* mean velocity, *AP* anterior–posterior, *ML* medial–lateral, *QC* quite stance-closed eyes

^*^Correlation is significant at the 0.05 level (2-tailed). ** Correlation is significant at the 0.01 level (2-tailed)

We would like to thank an anonymous reviewer for highlighting a slight school-grade difference, which was not significant between the control and the intervention group (see Table 1). Keeping in mind clarification on the teaching system (see more information in Section “Subjects and design” in the “Methods”) and in response to this suggestion, we ran further ANOVA analyses on children in grades 2 and 3, which produced the same pattern of significant results (Tables 5 and 6).

**Table 5:** Outcomes of mixed between-within ANOVA analyses for the sum of the second and third grade students, when the first-graders were excluded

Outcomes	Time^a^			Group^a^			Interaction		
	f	*p*-value	η_p_^2^	f	*p*-value	η_p_^2^	f	*p*-value	η_p_^2^
	(1–13)			(1–13)			(1–13)		
Clinical measures									
FDS	73.16	** < 0.001**	0.86	18.62	**0.001**	0.61	20.1	**0.001**	0.63
SCWT	14.1	**0.003**	0.54	0.29	0.603	0.02	0.42	0.531	0.03
WR	45.16	** < 0.001**	0.79	26.3	** < 0.001**	0.69	26.3	** < 0.001**	0.69
NWR	16.16	**0.002**	0.57	1.89	0.194	0.14	8.29	**0.014**	0.41
PD	56.34	** < 0.001**	0.82	0.9	0.37	0.07	17.12	**0.001**	0.59
TC	57.83	** < 0.001**	0.83	0.09	0.77	0.01	5.79	**0.033**	0.33
CW	22.54	** < 0.001**	0.65	1.19	0.296	0.09	10.07	**0.008**	0.46
CoP measures									
QO-L (cm)	0.22	0.647	0.02	3.62	0.081	0.23	1.47	0.25	0.2
QO-A (cm^2^)	1.94	0.189	0.14	1.07	0.321	0.08	16.46	**0.002**	0.58
QO-MV (cm/s)	0.25	0.628	0.02	3.56	0.084	0.23	1.41	0.258	0.11
QO-AP (SD)	0.24	0.639	0.02	1.17	0.301	0.09	10.05	**0.004**	0.27
QO-ML (SD)	10.05	**0.008**	0.46	0.09	0.765	0.01	16.84	**0.001**	0.58
QC-L (cm)	1.9	0.195	0.14	0.78	0.396	0.06	0	0.993	0
QC-A (cm^2^)	0.22	0.644	0.02	0.38	0.551	0.03	1.8	0.204	0.13
QC-MV (cm/s)	2.36	0.151	0.16	0.93	0.353	0.07	0.03	0.864	0
QC-AP (SD)	0.03	0.866	0	0.08	0.783	0	6.27	**0.028**	0.34
QC-ML (SD)	2.23	0.161	0.16	0.08	0.782	0	0.03	0.861	0

Bolded values indicate statistically significant *p*-values (*p* < .05)

*FDS* forward digit span, *SCWT* Stroop color-word test, *WR* word reading, *NWR* non-word reading, *PD* phoneme deletion, *TC* text comprehension, *CW* chain word, *CoP* center of pressure, *QO* quite stance-open eyes, *L* length, *A* area, *MV* mean velocity, *AP* anterior–posterior, *ML* medial–lateral, *QC* quite stance-closed eyes

^a^Between-subjects factor ‘Group’ = control group vs. intervention group, and within-subjects factor ‘Time’ = measurement at before and after intervention

**Table 6:** Mean (standard deviation) of the clinical and center of pressure measures for the sum of the second and third grade students, when the first-graders were excluded

Outcomes	CG (n = 8)		IG (n = 7)		Total (N = 15)	
	Pre	Post	Pre	Post	Pre	Post
Clinical measures						
FDS	2.50 (0.53)	3.12 (0.64)	2.83 (0.41)	4.83 (0.41)	2.64 (0.50)	3.86 (1.02)
SCWT	192.50 (63.89)	160.00 (73.13)	180.00 (75.96)	134.00 (64.83)	187.14 (66.77)	148.86 (68.37)
WR	57.13 (27.94)	69.13 (26.62)	52.16 (29.99)	141.50 (65.28)	55.00 (27.80)	100.14 (58.33)
NWR	12.63 (4.41)	14.50 (4.84)	12.50 (6.19)	23.83 (11.43)	12.57 (5.02)	18.50 (9.26)
PD	16.90 (11.40)	20.25 (11.27)	17.50 (8.10)	29.17 (3.77)	17.14 (9.74)	24.10 (9.74)
TC	3.90 (1.90)	5.00 (2.27)	3.67 (1.86)	5.83 (1.60)	3.79 (1.81)	5.36 (1.98)
CW	17.25 (6.19)	21.63 (9.04)	13.67 (8.64)	35.67 (1.17)	15.71 (7.26)	27.64 (14.02)
CoP measures						
QO-L (cm)	119.76 (45.47)	126.84 (50.93)	96.15 (21.72)	80.10 (11.10)	109.64 (37.10)	106.80 (45.05)
QO-A (cm^2^)	6.57 (3.85)	8.48 (4.89)	7.57 (3.50)	3.67 (1.85)	7.00 (3.28)	6.42 (4.50)
QO-MV (cm/s)	2.00 (0.76)	2.11 (0.85)	1.60 (0.36)	1.30 (0.20)	1.83 (0.63)	1.80 (0.75)
QO-AP (SD)	0.61 (0.22)	0.76 (0.35)	0.67 (0.22)	0.46 (0.08)	0.64 (0.21)	0.63 (0.30)
QO-ML (SD)	0.55 (0.21)	0.58 (0.20)	0.64 (0.16)	0.43 (0.20)	0.59 (0.19)	0.51 (0.21)
QC-L (cm)	144.39 (37.69)	135.81 (33.77)	130.23 (20.05)	121.76 (29.53)	138.32 (31.18)	129.79 (31.65)
QC-A (cm^2^)	8.96 (5.56)	9.80 (5.70)	8.75 (3.65)	7.02 (3.13)	8.87 (4.65)	8.61 (4.81)
QC-MV (cm/s)	2.41 (0.63)	2.30 (0.56)	2.17 (0.33)	2.00 (0.46)	2.31 (0.52)	2.15 (0.52)
QC-AP (SD)	0.72 (0.23)	0.86 (0.28)	0.84 (0.32)	0.68 (0.14)	0.77 (0.27)	0.78 (0.24)
QC-ML (SD)	0.62 (0.29)	0.56 (0.21)	0.58 (0.12)	0.53 (0.14)	0.60 (0.22)	0.55 (0.18)

*FDS* forward digit span, *SCWT* Stroop color-word test, *WR* word reading, *NWR* non-word reading, *PD* phoneme deletion, *TC* text comprehension, *CW* chain word, *CoP* center of pressure, *QO* quite stance-open eyes, *L* length, *A* area, *MV* mean velocity, *AP* anterior–posterior, *ML* medial–lateral, *QC* quite stance-closed eyes, *CG* control group, *IG* intervention group, *Pre* pre-intervention, *Post* post-intervention

## Discussion

In the current research, we aimed to examine the effectiveness of the VWM-B program on verbal WM capacity, reading ability, and postural control in children with DD. In comparison with VWM-program, the VWM-B program showed superior performance on verbal WM capacity, reading skills, and postural control after a short-term intervention.

Based on our best knowledge, VWM-B is the only training program, which contains a mix of cognitive and balance-related performance simultaneously, which has been used in DD. Previous studies have also reported some positive effects for a combination of cognitive and physical training in other populations [82–84]. Regarding the sequential nature of the process in a dual-task condition, e.g., the VWM-B program, the nervous system first prioritizes a task and then assigns further cognitive/attentional resources for the prioritized task. Therefore, the performance decreases on the non-priority task [85]. Furthermore, sufficient manipulating and maintaining information in the verbal WM is critical for increasing verbal WM capacity [86]. Keeping these points in mind, the featured maintenance step of the VWM-B program probably had an important role in efficiently improving the measured functions in children with DD (see Section “VWM-B program for participants in the intervention group” in the “Methods”). We designed the maintenance step of the VWM-B program within two passive and active balance states of the subject. In the passive state, we designed the cognitive task as a prioritized task. In the active state, however, the balance was considered a prioritized task. Hence, we expected the balance-related movements would be automatized [75], and as a result, further resources assign to the cognitive task [85]. The cerebellar deficit hypothesis in dyslexia characterizes the behavioral symptoms of dyslexia as difficulties in skills automatization [16]. Based on this theory, the cerebellum is a key structure in the automatization deficits [87]. Therefore, it appears that the positive effects of the VWM-B program on the measured functions stemmed from its effects on cerebellum activation.

The present study showed improvement in the intervention group participants’ postural control after the intervention, which was perceived in both eyes open and closed conditions of CoP. It indicates that the balance-related movements were automatized after the intervention. The older evidence revealed that there is no significant difference between the upright standing postural control (eyes open) of the dyslexic and non-dyslexic children [88]. However, children with DD have weaker postural control when they use visual information to perform an activity (actions often are complex or dual-task) [34, 88]. The reason is supposedly insufficient coupling of the visual inputs and postural sway while performing an activity [88]. The improved postural control in the eyes-open condition demonstrates that coupling visual information and body sway were probably improved, and the intervention group participants could assign sensory information to produce purposeful actions [88]. In other words, these participants showed higher performance in using non-visual information to maintain postural control and benefit from the visual information to perform purposeful (cognitive) actions. The improved postural control in the eyes-closed condition implies that the intervention group participants probably benefited from vestibular and/or proprioceptive information and were less dependent on visual inputs to maintain postural control [89]. Since there were no significant changes in the CoP measures of the control group participants, it is concluded that the motor strategies relating to balance control were automatized [75, 88, 90, 91], and further neural resources were allocated to the cognitive task following the intervention by the VWM-B program [85, 90, 91].

In the present study, participants who had received training by the VWM-B program showed higher performance in verbal WM and reading skills. Also, the current research demonstrated that improved postural control was correlated with verbal WM and reading ability. Furthermore, verbal WM was correlated with reading ability. Although the current research has emphasized the cerebellar deficits hypothesis to interpret the results, the causal link between the balance deficits and reading problems is still under controversy [74, 92–94]. On the other hand, literature has confirmed the critical role of the cerebellum in verbal WM deficits [32]. Hence, it appears that the automatized postural control initially caused an improvement in verbal WM capacity; and thereupon, the increased verbal WM capacity has led to an improvement in reading ability [95]. It seems that the improved verbal WM capacity facilitated the word and non-word reading ability via improved phonological awareness (tested by the WR and phoneme deletion subtests of NEMA) [96, 97]. Also, improvement in the grapheme–phoneme decoding (tested by the NWR and word chain subtests of NEMA) and phonemic awareness (tested by the WR and phoneme deletion subtests of NEMA) may be related to an improvement in reading comprehension (tested by the text comprehension subtest of NEMA) [97, 98]. In the case of attention, the close link between WM capacity and attention has been confirmed by previous studies [99]. It has been reported that the WM modulates attention [69, 99, 100], and on the other hand, that attention promotes the encoding, maintaining, and manipulating of information in the WM [101, 102]. Despite these pieces of evidence, the Stroop test outcomes in the present study were uncorrelated with verbal WM capacity. Decreasing in the Stroop interference was correlated only with the WR subtest of NEMA. The significantly decreased Stroop interference in the intervention group participants could be justified by the structure of Stroop test. The word-naming step of the Stroop needs the subject to read the colored words [68]. It shows the subjects’ reading rate and reflects their speech-motor problems [68, 71]. Children with DD usually spend further time to complete this component [68, 70]. Keeping this point in mind, the decreased Stroop interference in the current study probably resulted from the improvement in word reading ability (tested by the WR subtest of NEMA). This claim is supported by previous studies when they declared that decreased Stroop interference implies improvements in reading ability as well as selective attention [68–70].

The cerebellar deficit hypothesis has also emphasized the impairments in the procedural learning system, with specific deficits in the language/cerebellar procedural circuits [87]. Almost all human activity needs trial-and-error (supervised) learning, which is a sub-type of procedural learning [87]. The cerebellum is a central structure in human brain circuits, and it is a crucial point that only the cerebellum has a hub circuitry to support supervised learning [87]. It implies that if this type of learning is required (e.g., in reading), it is necessary to involve the cerebellum as part of the circuit, along with the other parts of the brain involved in reading [87] (e.g., cortical regions of perisylvian [6] and prefrontal [23] involved in phonological processing and verbal WM, respectively). The corticocerebellar circuits involve loops from the cortex to the cerebellum to thalamic nuclei and back to the cortex [103]. Insufficient skill automatization due to impaired cerebellar function leads to problems in reading, though via different cerebellar circuits [104]. Considering the findings of the current study for CoP, it seems that the VWM-B program caused changes in the activation of cerebellum circuitry. Some regions of the cerebellum may be activated in this dual-task performance [36] and could integrate motor and cognitive networks and adjust these networks to be more efficient for performing the dual-task properly [36].

Although it needs future neuroimaging studies to adequately investigate the changes in the activation of the cerebellar circuits after treatment with the VWM-B program, previous neuroimaging studies have confirmed the role of the cerebellum in verbal WM, reading, balance, and complex actions [105, 106]. For example, a loop between the right VI and crus I lobules of the cerebellum and Broca's region of the left frontal lobe activates during articulatory rehearsal and verbal WM tasks [105, 107]. Activation of the right VI and crus I lobules of the cerebellum provide internal motor sequences for the phonological content of words [106]. Also, it has been reported that the loops between the bilateral cerebellar VI and VII lobules and the cerebral regions of the left inferior frontal lobe and the left inferior occipitotemporal lobe have a critical role in the reading network [108, 109]. Furthermore, researchers have recently discovered a novel topographic map in the cerebellar lobules of VI and VIIA, which shows the role of these lobules in complex motor tasks [105]. Therefore, the authors suggest considering these cerebellar regions in future neuroimaging studies by treatment with the VWM-B program.

There are limited studies that investigate balance training effects on children with DD. For example, Goulème et al. demonstrated the effect of balance training only on postural control [28]; however, Reynolds et al. reported the positive effects of the exercise‐based treatment on balance, dexterity, eye movement control, and cognitive skills underlying literacy [110]. Whereas Rack et al. [111], following a commentary on the Reynolds et al. study [110, 111], have not confirmed the reported results. Overall, there were no sufficient balance training methods to improve the balance and literacy in the children with DD. Therefore, the present study has introduced a newly designed training program, for the first time, which has positive effects concurrently on postural control and reading-related cognitive functions in children with DD. However, this study has some limitations. Various differences in the quality of educational services may be observed between different districts in Tehran as a metropolis. Regarding the socio-economic status of participants as an inclusion criterion, study participants were recruited from the public primary schools located in District 20, Tehran, Iran. Therefore, recruitment did not include students of private schools located in this region because of possible different educational services. Moreover, the present study investigated only the short-term effects of the VWM-B program, and its long-term effectiveness needs to be followed up in the future. We suggest investigating the effectiveness of the VWM-B program on attention using suitable measurements such as eye-tracking studies to investigate visual attention [112] and eye-movement changes, especially fixation [113] as an indicator for improving attention in DD. Also for future studies, a dyslexia control group without interventions should be considered.

## Conclusions

The present study is a pioneer in investigating the effectiveness of a newly structured VWM-B training program. This program provides a dual-task condition including cognitive (verbal WM and reading) and motor (passive and active balance state) tasks. This study demonstrated that the VWM-B program, after the short-term treatment, is more effective than the VWM-program in the improvement of verbal WM capacity, reading skills, and postural control in the children with DD. The improvement in postural control (automatization in the balance-related movements) probably had an effective role in improving the measured cognitive functions. It seems that the automatization in balance-related movements consequently led to assigning further neural resources to the cognitive task. The Cerebellum has a critical role in maintaining postural control and automatizing skills; therefore, the activation of the cerebellum regions may be changed after the intervention by the VWM-B program. Despite the results of the current studies that support the cerebellar deficits hypothesis, the role of the cerebellum in DD is still controversial.

## Supplementary Information

**Additional file 1: Appendix S1.** CONSORT checklist.

## Data Availability

The data analyzed during the current study are available from the corresponding author on reasonable request.

## Abbreviations

WM: Working memory
VWM-B: Verbal working memory-balance
WISC-IV: Wechsler intelligence scale for children-fourth edition
CSI-4: Child symptoms inventory-four
CoP: Center of pressure
NEMA: Persian battery of normative reading tests
WR: Words reading
NWR: Non-words reading
FDS: Forward digit span
