# Supplementary material for: Evaluating a new verbal working memory-balance program: a double-blind, randomized controlled trial study on Iranian children with dyslexia
Source: BMC Neurosci. 2021 Sep 15;22:55. doi: 10.1186/s12868-021-00660-1 (PMC8442443; doi:10.1186/s12868-021-00660-1)
Supplement: Supplementary file 1 — Additional file 1: Appendix S1. CONSORT checklist. [file 12868_2021_660_MOESM1_ESM.pdf]

Appendix S1. CONSORT checklist

Section/Topic	Item No	Checklist item	Reported on page No
**Title and abstract**			
	1a	Identification as a randomized trial in the title	1
	1b	Structured summary of trial design, methods, results, and conclusions (for specific guidance see CONSORT for abstracts)	2
**Introduction**			
**Background and objectives**	2a	Scientific background and explanation of rationale	3
	2b	Specific objectives or hypotheses	3
**Methods**			
**Trial design**	3a	Description of trial design (such as parallel, factorial) including allocation ratio	4
	3b	Important changes to methods after trial commencement (such as eligibility criteria), with reasons	N / A
**Participants**	4a	Eligibility criteria for participants	4
	4b	Settings and locations where the data were collected	4
**Interventions**	5	The interventions for each group with sufficient details to allow replication, including how and when they were actually administered	5-6
**Outcomes**	6a	Completely defined pre-specified primary and secondary outcome measures, including how and when they were assessed	6-7
	6b	Any changes to trial outcomes after the trial commenced, with reasons	N /A
**Sample size**	7a	How sample size was determined	4
	7b	When applicable, explanation of any interim analyses and stopping guidelines	N / A
**Randomization:**			
**Sequence generation**	8a	Method used to generate the random allocation sequence	4
	8b	Type of randomization; details of any restriction (such as blocking and block size)	4
**Allocation concealment mechanism**	9	Mechanism used to implement the random allocation sequence (such as sequentially numbered containers), describing any steps taken to conceal the sequence until interventions were assigned	4
**Implementation**	10	Who generated the random allocation sequence, who enrolled participants, and who assigned participants to interventions	4
**Blinding**	11a	If done, who was blinded after assignment to interventions (for example, participants, care providers, those assessing outcomes) and how	5

	11b	If relevant, description of the similarity of interventions	6
**Statistical methods**	12a	Statistical methods used to compare groups for primary and secondary outcomes	7
	12b	Methods for additional analyses, such as subgroup analyses and adjusted analyses	N / A
**Results**			
**Participant flow (a diagram is strongly recommended)**	13a	For each group, the numbers of participants who were randomly assigned, received intended treatment, and were analyzed for the primary outcome	Figure 1
	13b	For each group, losses and exclusions after randomization, together with reasons	Figure 1
**Recruitment**	14a	Dates defining the periods of recruitment and follow-up	Figure 1
	14b	Why the trial ended or was stopped	N / A
**Baseline data**	15	A table showing baseline demographic and clinical characteristics for each group	Tables 1, 2, and 6
**Numbers analyzed**	16	For each group, number of participants (denominator) included in each analysis and whether the analysis was by original assigned groups	Figure 1
**Outcomes and estimation**	17a	For each primary and secondary outcome, results for each group, and the estimated effect size and its precision (such as 95% confidence interval)	Table 2, 3, 5, and 6
	17b	For binary outcomes, presentation of both absolute and relative effect sizes is recommended	N / A
**Ancillary analyses**	18	Results of any other analyses performed, including subgroup analyses and adjusted analyses, distinguishing pre-specified from exploratory	Table 4
**Harms**	19	All important harms or unintended effects in each group (for specific guidance see CONSORT for harms)	N / A
**Discussion**			
**Limitations**	20	Trial limitations, addressing sources of potential bias, imprecision, and, if relevant, multiplicity of analyses	12
**Generalizability**	21	Generalizability (external validity, applicability) of the trial findings	12
**Interpretation**	22	Interpretation consistent with results, balancing benefits and harms, and considering other relevant evidence	11-12
**Other information**			
**Registration**	23	Registration number and name of trial registry	13
**Protocol**	24	Where the full trial protocol can be accessed, if available	13
**Funding**	25	Sources of funding and other support (such as supply of drugs), role of funders	13
